# Correlation of Lipid Profile and Glycated Hemoglobin as a New Prognostic Criterion for Type 2 Diabetes Mellitus Development and Progression

**DOI:** 10.17691/stm2020.12.2.11

**Published:** 2020

**Authors:** E.A. Zagrebin, E.A. Shevchenko, E.Yu. Ivanchenko, V.I. Uspensky, S.R. Abasnia, A.V. Kochetkova, O.A. Uspenskaya, A.R. Vaysberg

**Affiliations:** PhD Student, Department of Pathological Physiology, Privolzhsky Research Medical University, 10/1 Minin and Pozharsky Square, Nizhny Novgorod, 603005, Russia;; Professor, Department of Pathological Physiology, Privolzhsky Research Medical University, 10/1 Minin and Pozharsky Square, Nizhny Novgorod, 603005, Russia;; Associate Professor, Department of Therapy and Cardiology, Privolzhsky Research Medical University, 10/1 Minin and Pozharsky Square, Nizhny Novgorod, 603005, Russia;; Student, National Research University Higher School of Economics, 25/12 Bolshaya Pecherskaya St., Nizhny Novgorod, 603005, Russia;; Assistant, Department of Pediatric Surgery, Anesthesiology and Intensive Care, Urgench Branch of the Tashkent Medical Academy, 28 Al-Khorazmi St., Ugench, Khorezm region, 220100, Uzbekistan;; Student, Privolzhsky Research Medical University, 10/1 Minin and Pozharsky Square, Nizhny Novgorod, 603005, Russia;; Associate Professor, Head of the Department of Therapeutic Dentistry, Privolzhsky Research Medical University, 10/1 Minin and Pozharsky Square, Nizhny Novgorod, 603005, Russia;; Associate Professor, Department of Therapy and Cardiology, Privolzhsky Research Medical University, 10/1 Minin and Pozharsky Square, Nizhny Novgorod, 603005, Russia

**Keywords:** lipid profile, glycated hemoglobin, metabolic syndrome, type 2 diabetes mellitus

## Abstract

**The aim of the study** was to assess correlations between the lipid profile and glycated hemoglobin in order to define their prognostic value as a criterion for the development and progression of type 2 diabetes mellitus (DM2) in individuals with metabolic syndrome and low body mass.

**Materials and Methods:**

The study included the results of examining 50 patients with metabolic syndrome (group 1), 50 with low body mass index (BMI) (group 2), 50 with DM2 (group 3), and 50 apparently healthy people (control group). Biochemical indices of the lipid profile and glycated hemoglobin in the venous blood were assessed using analyzers Clima MC-15 (Spain), BS-200, and BS-200 E (China).

**Results:**

It has been established that correlations of the parameters such as high-density lipoproteins (HDL), low-density lipoproteins (LDL), urea, and creatinine with BMI are expressed statistically more significantly in women in groups 1 and 2 in comparison with group 3, while in men the reverse situation is observed. Besides, correlation of triglyceride levels with BMI is statistically more marked in patients with DM2 than in apparently healthy people being positive in women and negative in men.

Thus, HDL, LDL, creatinine, and urea may be diagnostically significant in the assessment of the development and progression of DM2 in men with metabolic syndrome since a strong positive correlation has been found between them and BMI, triglycerides were also found to be significant because a strong negative correlation has been detected between them and BMI.

For women with metabolic syndrome, the combination of a weak correlation of BMI with the levels of HDL, LDL, urea, and creatinine and a strong positive correlation with triglycerides may be considered to be a prognostically significant index.

**Conclusion:**

New correlation characteristics of biochemical blood indices in DM2 may serve as prognostic criteria for disease development and progression.

## Introduction

In adults with diabetes, risk of infarction and stroke increases 2–3 times. In combination with blood flow reduction, neuropathy (nerve damage) of lower extremities increases the probability of appearing ulcers on the legs, their infection, and, ultimately, the necessity of limb amputation. Diabetes is included into the list of the main causes of renal failure, and general risk of death in these people is at least 2 times higher than in individuals of the same age without diabetes [1, 2].

According to WHO prognoses, diabetes may occupy the seventh place among the causes of death in 2030. Rigid control of the glucose level has fallen short of expectations, therefore, there is a strong necessity to control other parameters as well [3].

In this connection, it may be interesting to investigate the changes in the indices of the lipid profile and glycated hemoglobin and their interconnections with the body mass index (BMI) and with metabolic syndrome (MS) in patients with type 2 diabetes mellitus (DM2).

Though there is a great number of works devoted to this problem, questions of etiopathogenesis have not been fully studied, therefore, the number of people with MS and diabetes mellitus is steadily growing whereas traditional methods of treatment are not always sufficiently effective. As DM2 occurs not only in individuals with an increased BMI, it was interesting to know how these indices are changing in those with low BMI. We did not find any data on this question in the available literature [4–6]. The importance of studying the changes in the lipid profile indices to gain insights into pathogenesis of this disease has been proved for various disorders [7–9].

**The aim of the study** was to define the presence and assess the correlation dependences between the lipid profile and glycated hemoglobin in individuals with metabolic syndrome and with a low body mass as well as in type 2 diabetes mellitus.

## Materials and Methods

The study was carried out at the Department of Pathological Physiology and Department of Therapy and Cardiology of Privolzhsky Research Medical University (Nizhny Novgorod). The study included the results of examining 50 patients with metabolic syndrome (group 1), 50 with a low body mass index (BMI) (group 2), 50 with DM2 (group 3), and 50 apparently healthy people (control group). The diagnoses were established by therapeutists and endocrinologists according to the diagnostic criteria of the International Classification of Diseases (ICD-10). The participants underwent clinical and instrumental examination. Biochemical indices of the lipid profile and glycated hemoglobin were assessed using analyzers Clima MC-15 (RAL, Spain), BS-200, and BS-200 E (Mindray, China).

The study complied with the Declaration of Helsinki (2013) and was approved by the Ethics Committee of Privolzhsky Research Medical University. Written informed consent was obtained from every patient.

Venous blood was collected from all participants to define the levels of the lipid profile and glycated hemoglobin. Glycated hemoglobin was assessed according to the WHO standards: values up to 5.6% for a normal state, 5.7–6.4% is a high level connected with the risk of diabetes development, 6.4% and higher are values for diabetes mellitus.

Identification of glycated hemoglobin has an advantage over the usual blood test for glucose as it makes it possible not only to diagnose the glucose level over a sufficiently long period of time, i.e. about 3 months which corresponds to the erythrocyte lifetime of 90–120 days, but also to trace the dynamics of its changes by finding the average daily levels of glucose in the body over the given period.

Metabolic syndrome is a multifactorial complex of pathological changes with insulin resistance lying at its basis. The information about the combination of insulin resistance, arterial hypertension, hyperlipidemia, and obesity appeared as early as the end of the 20^th^ century. One of the difficult tasks for studying this syndrome was the choice of diagnostic criteria. Recommendations of the International Diabetes Federation (IDF) issued in 2005 are used most commonly. According to the data of the IDF consensus on MS, the main factors of the syndrome development are abdominal obesity and insulin resistance. In this connection, a patient is said to have MS if abdominal obesity is combined with two of the four factors: 1) blood triglyceride level of more than 1.7 mmol/L; 2) HDL level below 1.3 mmol/L in men and below 1.29 mmol/L in women; 3) arterial pressure over 130/85 mm Hg; fasting blood serum glucose level over 5.6 mmol/L [6]. The MS indices were taken for the study as criteria for DM2 diagnosis. These indices were compared between the three groups and then with the control values obtained for group 4.

**Statistical data processing** included determination of average values and standard deviations, analysis of data for normal distribution (asymmetry (A) and excess (E)), and was performed using methods of assessing the significance of the results, analysis of variance, detection, and assessment of correlations. The results were processed and analyzed using Microsoft Office (Excel) programs, a package of statistical programs Stada and Statistica v. 7.0, programming language R (RStudio 3.5.3.) and embedded and additional packages for processing and statistical analysis ggplot2, viridis, hrbrthemes.

## Results and Discussion

Metabolic syndrome often precedes the development of DM2 and, therefore, represents one of the main risk factors for cardiovascular diseases in modern society. Besides, it is associated with the development of hepatic steatosis, renal function disorder, and a high risk of oncological diseases [7].

Lipid profile and glycated hemoglobin have been investigated in four groups of patients: with MS, body mass deficit, DM2, and control. Correlations between the examined indices have been established and evaluated.

The analysis of the results obtained showed that correlations of the parameters such as the levels of high-density lipoproteins (HDL), low-density lipoproteins (LDL), urea, creatinine with BMI were expressed statistically much more significantly in women with MS (group 1). The reverse situation is observed in the men. Besides, the correlation of triglyceride levels with BMI in the patients with DM2 has been established to be expressed stronger than in the healthy persons, it being positive in the women and negative in men.

The revealed dependences signified the presence of multidirectional interconnections between the examined indices in all the groups. These interconnections were different for the examined men and women. These differences reflected the changes in the metabolic processes in the body in DM2 and without it and, consequently, may serve as a prognostic criterion for the development of this pathology in different cohorts of people both with MS and low BMI.

**Figure 1:**
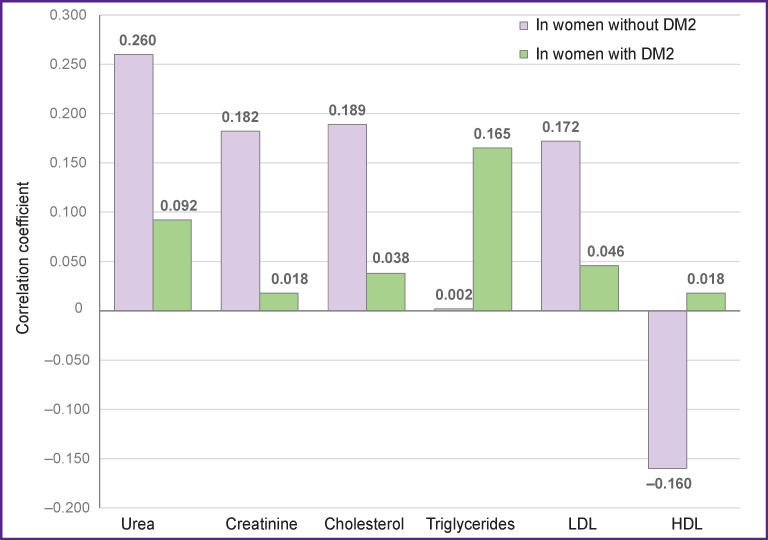
Correlations of lipid profile with BMI in the examined women Correlation coefficient: r>0.7 for a strong correlation; 0.3–0.7 medium correlation; <0.3 weak correlation; ***in women without DM2*** (groups 1 and 2): urea A=0.26, E=–0.72; creatinine A=0.4, E=0.02; cholesterol A=0.03, E=–0.2; triglycerides A=–1.26, E=–4; LDL A=0.34, E=0.6; HDL A=–1.2, E=0.33; p=0.008 according to the Kruskal–Wallis test; ***in women with DM2*** (group 3): urea A=0.14, E=0.61; creatinine A=–0.25, E=2.15; cholesterol A=–1.52, E=0.75; triglycerides A=0.75, E=2.35; LDL A=–1.22, E=0.66; HDL A=0.32, E=0.98; p=0.0012 according to the Kruskal–Wallis test

It has been found that strong positive correlations of LDL, cholesterol, creatinine, urea with BMI were observed in the women without DM2 (groups 1 and 2), whereas these correlations were weaker in the women with DM2 (Figure 1). A strong negative correlation of LDL with BMI was also detected in the women without DM2.

A different picture was observed for the women with DM2 (group 3). All correlations of the lipid profile with BMI were positive, weakly expressed, while the correlation between triglycerides and BMI was positive and strong. Therefore, the presence of strong correlations in this case may become a prognostic criterion of further DM2 development in these women.

**Figure 2:**
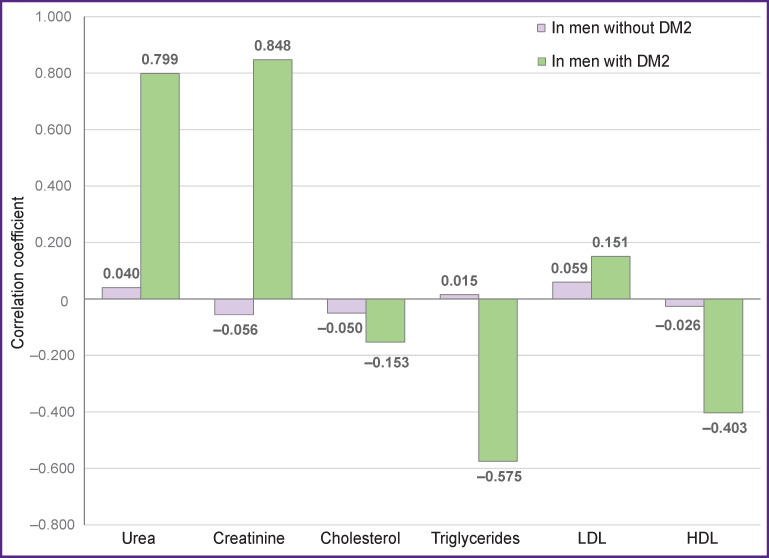
Correlations of lipid profile with BMI in the examined men Correlation coefficient: r>0.7 for a strong correlation; 0.3–0.7 medium correlation; <0.3 weak correlation; ***in men without DM2:*** urea A=1.47, E=0.95; creatinine A=1.14, E=–0.7; cholesterol A=0.37, E=0.35; triglycerides A=1.97, E=4.2; LDL A=1.05, E=0.07; HDL A=0.12, E=–2.97; p=0.0006 according to the Kruskal–Wallis test; ***in men with DM2:*** urea A=0.76, E=2.15; creatinine A=1.63, E=0.12; cholesterol A=1.11, E=–0.37; triglycerides A=0.1, E=0.17; LDL A=–1.08, E=0.31; HDL A=1.17, E=–1.05; p=0.0007 according to the Kruskal–Wallis test

Different relationships were found for the men. Correlations of HDL, LDL, urea, and creatinine with BMI were expressed stronger in the men with DM2 than in other groups (Figure 2).

**Figure 3:**
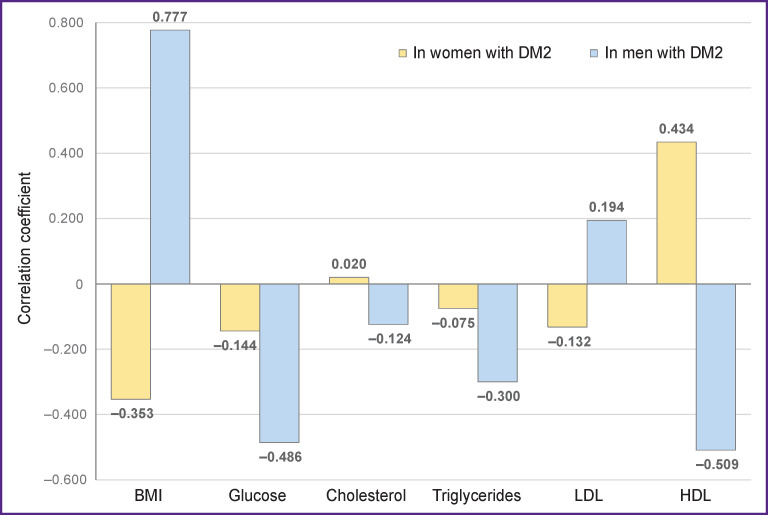
Correlations of glycated hemoglobin with BMI, glucose, and lipid profile in women and men with DM2 Correlation coefficient: r>0.7 for a strong correlation; 0.3–0.7 medium correlation; <0.3 weak correlation; ***in women:*** BMI A=1.15, E=0.73; glucose A=–0.17, E=0.93; cholesterol A=0.4, E=0.23; triglycerides A=1.02, E=0.46; LDL A=1.33, E=–0.23; HDL A=3.25, E=–1.62; p=0.002 according to the Kruskal–Wallis test; ***in men:*** BMI A=2.24, E=1.25; glucose A=1.44, E=0.73; cholesterol A=0.31, E=0.96; triglycerides A=2.73, E=–3.44; LDL A=0.76, E=–0.99; HDL A=1.16, E=–0.28; p=0.0045 according to the Kruskal– Wallis test

The analysis of the gender-differentiated results of correlation of glycated hemoglobin with the levels of BMI, glucose, and the lipid profile (HDL, LDL, triglycerides, cholesterol) in the patients with DM2 has shown that they were statistically significantly more expressed in the men than in women (Figure 3).

Thus, in men with MS and low BMI, it is necessary to pay special attention to the following biochemical indices important for the development and progression of DM2: HDL, LDL, creatinine, and urea as, according to the findings of the investigation, a strong correlation was found between them and BMI, attention should also be paid to triglycerides because a strong negative correlation was estimated between them and BMI.

For women with MS and low BMI, a combination of a weak correlation of BMI with HDL, LDL, creatinine, and urea and a strong positive correlation of BMI with triglycerides will be prognostically significant for the development and progression of DM2.

The results of the study also showed a multidirectional (opposite) correlation of BMI, cholesterol, HDL, LDL with glycated hemoglobin in men and women that should be taken into consideration when assessing the development of diabetes mellitus.

## Conclusion

New correlation characteristics of biochemical blood indices in DM2 may serve as prognostic criteria of the disease development and progression.

## References

[1] Ametov A.S., Barykina I.N., Bondar I.A., Vaysberg A.R., Verbovaya N.I., Zhukova L.A., Zamyatina O.V., Kiseleva T.P., Morugova T.V., Hostalec U.G., Shabalina E.A (2017). Adherence of patients to the metformin therapy with prolonged action (Glucophage® long) in real clinical practice in the Russian Federation.. Endokrinologiya: novosti, mneniya, obuchenie.

[2] Shevchenko E.A., Uspenskaya O.A., Kondyurov I.M., Kurylev V.V., Rossokhin V.F (2012). The estimation of a virus component for diagnosis and treatment of oral inflammatory diseases.. Sovremennye tekhnologii v meditsine.

[3] Valikulova F.Yu., Fomin I.V., Mudrova L.A., Vaysberg A.R. (2013). The state of control of hypercholesterolemia in patients with diabetes mellitus and coronary heart disease in an outpatient setting.. CardioSomatika.

[4] Zagoskin P.P., Zagoskina I.P., Savelieva N.A., Lyalyaev V.A (2014). Modern approaches to the problem of body weight regulation (review).. Sovremennye tekhnologii v meditsine.

[5] Ellaryan L.K., Kazarina L.N., Shevchenko E.A (2018). A complex approach to glossalgia treatment based on the current data on the specificity of its etiopathogenesis.. Sovremennye tekhnologii v meditsine.

[6] Kanygina E.L., Klemenova I.A., Kurnikov G.Yu. (1998). Lipid complex for the treatment of chronic dermatoses..

[7] Klemenova I.A., Kopytova T.V., Abalikhina E.P (2002). The study of the spectrum of lipids as a way of treating patients with psoriasis.. Klinicheskaya laboratornaya diagnostika.

[8] Shevchenko E.A., Artifeksova A.A., Uspenskaya O.A (2011). The role of urogenital infection in the mechanism of infertility development.. Sovremennye tekhnologii v meditsine.

[9] Shevchenko E.A., Zagrebin E.A., Chilipyonok A.S., Zaletina A.V., Uspenskij V.I., Ivanchenko E.Yu., Kochetkova A.V. (2019). Identification of risk factors of development of a dysbiosis in persons of young age with a metabolic syndrome and without it by means of information and computer technologies.. Medicinskij al’manah.

